# Correlation of Various Techniques in Diagnosis of Tuberculous Lymphadenitis on Fine Needle Aspiration Cytology

**DOI:** 10.1155/2013/824620

**Published:** 2013-09-19

**Authors:** Brijesh Thakur, Ravi Mehrotra, Jitendra Singh Nigam

**Affiliations:** ^1^Department of Pathology, SGRRIM & HS, Patel Nagar Industrial Area, Niranjanpur, Dehradun, Uttarakhand 248001, India; ^2^Institute of Cytology and Preventive Oncology (ICPO), I-7 Sector 39, Noida, Uttar Pradesh 201303, India; ^3^Department of Pathology, Deen Dayal Upadhyay Hospital, Harinagar, New Delhi 110066, India

## Abstract

*Objective*. To study the correlation of cytomorphological features in fine needle aspiration smears from patients suspected of having tuberculous lymphadenitis with Ziehl-Neelsen staining (ZN), auramine-rhodamine staining (ARS), and autofluorescence (AF). *Methods*. A total of 145 lymph nodes were aspirated, 3 air-dried smears were stained with Giemsa, Ziehl-Neelsen, and auramine-rhodamine stains, and 1 smear was wet fixed for Papanicolaou staining. Needle washes were incubated in Lowenstein-Jensen medium for culture. Papanicolaou and auramine-rhodamine stained smears were examined under fluorescent microscope using a blue excitation filter (450–480 nm). *Results*. Ninety aspirates were reported on cytomorphology as suggestive of tuberculous lymphadenitis. Smear positivity for *Mycobacteria* by Ziehl-Neelsen method was 26.67% (24/90), while positivity increased to 34.44% (31/90) by auramine-rhodamine and 42.22% (38/90) on autofluorescence. Culture was positive in 27.78% (25/90) aspirates. Using culture as the reference method, the statistical values of ZN, ARS, and AF were as follows: sensitivity 80.0%, 88.0%, 96.0%; specificity 93.85%, 86.15%, 78.46%; positive predictive values 83.33%, 70.97%, 63.16%; and negative predictive values 92.42%, 94.92%, 98.08%, respectively. *Conclusion*. There is a definite advantage of autofluorescence over Ziehl-Neelsen and auramine-rhodamine which is to detect *Mycobacteria*, being more sensitive as well as an inexpensive technique. Autofluorescence can be a useful addition to routine cytology for early diagnosis and effective treatment.

## 1. Introduction

Tuberculosis (TB) is a major health problem in developing countries. Lymphadenopathy is the most common presentation of extrapulmonary tuberculosis [1, 2]. The precise cause of these enlarged lymph nodes is often difficult to establish by history, physical examination, and radiographic studies alone. Fine needle aspiration cytology (FNAC) has assumed an important role in the evaluation of peripheral adenopathy as a possible noninvasive alternative to excisional biopsy [3].

The cytological criteria for the diagnosis of possible tubercular lymphadenitis have been clearly defined as epithelioid cell granulomas with or without multinucleated giant cells and caseation necrosis [4]. Culture is essential for a definitive diagnosis; however, it takes weeks for identification, and its sensitivity is also relatively low in paucibacillary conditions [5, 6]. Conventional Ziehl-Neelsen (ZN) method for acid-fast bacilli (AFB) plays a key role in the diagnosis and the monitoring of treatment in tuberculosis [7]. Its major disadvantages are low sensitivity, time consuming, and oil immersion use.

Fluorescent microscopy plays an important role for detection of *Mycobacteria* because lower magnifications are used as well as less time is required to examine smears. Fluorescence microscopy using auramine-rhodamine (AR) or Papanicolaou (PAP) staining has been considered to be superior to ZN staining [8, 9]. The method is quick and inexpensive, and it can be focused on those specimens felt to harbour the above infections on morphologic grounds. This suggests that it can provide a rapid, safe, and inexpensive technique for early provisional diagnosis of mycobacterial infection in cytological specimens, but it shows proclivity towards observer bias and problems associated with artifacts.

Basically, the study was an attempt to find out cost-effective, rapid, and sensitive technique which can be used routinely in developing countries for early diagnosis and effective treatment of tuberculous lymphadenitis. The study demonstrated the correlation of the cytomorphological features with various techniques in FNA smears from patients who are suspected of having tuberculous lymphadenitis. We tried to use fluorescent microscopy (auramine-rhodamine staining (ARS) and autofluorescence (AF)) to detect *Mycobacterium* and to compare it with conventional ZN method on lymph node aspirates in cytology.

## 2. Material and Methods

A total of 145 patients suspected clinically of having tuberculosis with peripheral lymphadenopathy were referred for FNAC to the cytology unit of the Department of Pathology, from August, 2010 to July, 2011. A pretested proforma was used for collection of demographic information, relevant clinical history, and physical examination findings of each patient. Routine investigations including hemogram, mantoux test, and chest radiogram were performed. 

Both males and females (>1 yr) with well palpable and enlarged peripheral lymph node were included. Patients with multiple enlarged lymph nodes were enrolled, but a separate study form was not used for each lymph node. Exclusion criteria were age <1 year, very small or nonpalpable lymph nodes, or known cases of malignant, allergic, or skin disorders.

Four smears were made from each aspirate: three air-dried smears were stained with Giemsa, ZN, and AR stains and one was wet fixed for PAP stain for autofluorescence. Needle washes were incubated for culture over Lowenstein-Jensen medium at the same time. If aspirate was found to be inadequate, FNA was repeated at the same time for better retrieval of aspirate. Culture over Lowenstein-Jensen medium was taken as a reference method. PAP and AR stained slides were examined under fluorescent microscope using the blue excitation filter (450–480 nm). *Mycobacteria* appear as greenish yellow, slender, and slightly curved rod-shaped (400X). ZN stained smears were examined for AFB under oil immersion (1000X) using light microscopy which appeared as pinkish, thin curved rod-shaped bacterium measuring 0.5 to 3 micrometer and sometimes as beaded.

## 3. Results

**Figure 1:**
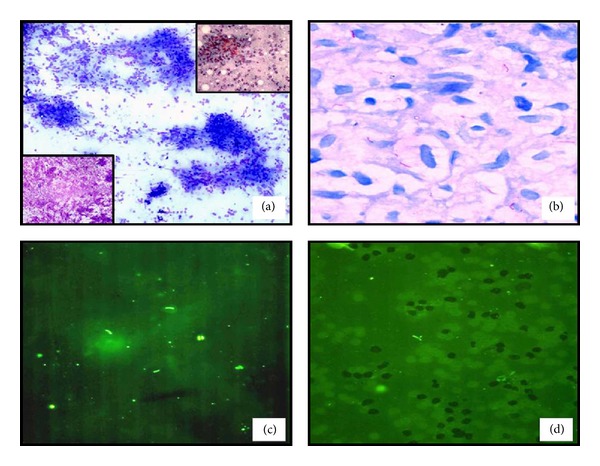
(a) Tuberculous lymphadenitis showing many well-formed epithelioid cell granulomas (Giemas ×100) with Caseating tuberculous lymphadenitis (left lower inset, Giemas ×400) and granuloma along with numerous neutrophils (right upper inset PAP, ×400). (b) Acid fast bacilli (arrows) in a case of tuberculous lymphadenitis (ZN ×1000). (c) Demonstration of bacilli through autofluorescence technique on Papanicolaou stained smears (Pap ×400). (d) Demonstration of bacilli on Auramine-rhodamine stained smear under fluorescent microscopy using blue excitation filter (Auramine-rhodamine ×400).

Out of the 145 cases, 90 (62.1%) aspirates were reported as cytomorphology suggestive of tuberculous lymphadenitis. Rest of the aspirates showed either blood or a reactive population of lymphoid cells only. Culture was also negative for these aspirates. Mantoux test was positive in 64.4% (58/90) cases, but significantly positivity (more than 10 mm.) was in 38.8% of the cases. Chest radiograms showed features of pulmonary tuberculosis in 32.2% (29/90) cases. Depending upon cytomorphological features, three patterns were found in tuberculous lymphadenitis: (1) granulomatous lymphadenitis, (2) caseating necrotising lymphadenitis, and (3) acute inflammation with granulomas. The cytomorphological features observed were granulomatous lymphadenitis in 57.8% (52/90), caseating necrotising lymphadenitis in (left lower inset Figure 1(a)) 31.1% (28/90), and acute inflammation with granuloma (right upper inset Figure 1(a)) in 11.1% (10/90) cases. (Figure 1(a)).

The age ranged from 2–60 years. Sixty% (54/90) of the cases with suggestive cytomorphology of tubercular lymphadenitis were in the range of 10–30 years of age. Male preponderance was noted accounting for 61.1% (55/90) of cases. In this study, the most common site of involved lymph nodes was of the cervical region in 83.3% (75/90) of the cases. On the basis of appearance of aspirate, blood mixed aspirates were found more commonly in 51.1% cases (46/90), followed by pus in 31.1% (28/90), cheesy white material in 15.6% (14/90), and clear fluid in 2.2% (2/90). Smear positivity for *Mycobacteria* by conventional ZN method was 26.7% (24/90), while positivity increased to 34.4% (31/90) by ARS and to 42.2% (38/90) on AF (Figures 1(b), 1(c), and 1(d)). Comparative results of positivity for *Mycobacteria* in various appearances of aspirates and cytomorphological patterns are shown in Table 1. Culture was positive in 27.8% (25/90) cases with suggestive cytomorphology of tubercular lymphadenitis. Using culture as reference method, a comparative chart of ZN, AR, and AF in cases that were reported as cytomorphology suggestive of tuberculous lymphadenitis was shown in Table 2, and the statistical values of ZN, AR, and AF were compared in Table 3.

## 4. Discussion

FNAC is an easy, reliable outpatient procedure for the diagnosis of tubercular lymphadenitis in palpable superficial lymph nodes, and it is ideally suited for use in resource limited settings, especially in developing countries where tuberculosis is a major cause of morbidity and mortality [10]. Several conditions, including mycosis, bacterial, and viral adenitis, can present the same cytology as does *Mycobacterium* Tubercular adenitis does. Laboratory tests may be essential to establish the cause of such adenopathy correctly, because treatment and prognosis may differ. Demonstration of *Mycobacterium* tuberculosis in fine needle aspirates becomes necessary for an early and accurate treatment. Directed treatment may decrease morbidity and prolong life expectancy.

Auramine-rhodamine involves the use of toxic and carcinogenic substances, but it requires less time, low power examination and shows superiority to ZN staining as auramine combined more readily with mycolic acid than the conventional carbol-fuchsin acid-fast stain. In comparison to both, AF on PAP stained smears provides a safe, inexpensive, and exposure-free diagnostic procedure along with more positive results. Even this technique does not require any addition to the standard PAP stain. Single PAP stained smear can be used for routine light microscopy to determine cytological diagnosis and for demonstrating *Mycobacteria* using fluorescent microscope. *Mycobacteria* AF, also known as primary fluorescence, is seen as brilliant yellowish green bacilli, thin, and slightly curved with polar enhancement and sometimes even beaded appearance.

Previous studies for detection of AFB from various clinical specimens, comprising sputum, CSF, fine needle aspirate, pus, and miscellaneous body fluids which were examined by ZN and AR staining techniques, showed that AR was 86.6% sensitive as compared to ZN 67.3% sensitive with more marked difference in extrapulmonary samples [8]. In other studies on FNA smears of lymph nodes, autofluorescence was found to be more sensitive than ZN staining [11, 12], but as compared to cytodiagnosis, it was less sensitive [9].

The present study was an attempt to use fluorescent microscopy (ARS and AF) to detect *Mycobacterium* and to compare it with conventional ZN method on lymph node aspirates in cytology. During the present study by using culture as the reference method, true statistical values of these techniques were given in Table 3. By these results, AF was noted to be more sensitive in identifying the acid fast bacilli than ZN stain; however, ZN was found to be more specific than AF.

A comparison between AF and ARS was also done in the present study. It was found that autofluorescence was more sensitive for identifying the bacilli. There was only one case (1.1%) which showed ARS positivity on smear but was negative by AF. However, 8 cases (8.89%) were AF positive, but they were found to be ARS negative. No comparative study between autofluorescence and auramine-rhodamine staining on FNAC smears could be found in literature searched.

However, some organisms exhibiting spontaneous emission spectra following excitation at specific wavelengths may pose problem in the detection of *Mycobacteria*. These organisms such as spore-forminglike *Bacillus subtilis*, nonspore-forming bacteria like *Staphylococcus aureus*, *Nocardia*, budding yeast may produce fluorescence [13]. Even air-drying artifacts in Papanicolaou stained smears may produce problems in identifying *Mycobacteria* by fluorescent microscopy. There is also increased frequency of false positivity of AF due to subjective biased errors and interobserver's variability.

## 5. Conclusion

FNAC as a primary diagnostic procedure in tubercular lymphadenitis has demonstrated the adaptability of autofluorescence on Papanicolaou stain which is the most frequently utilized stain for cytologic specimens and autofluorescence on the Pap smear is a rapid and relatively simple method. This study explores the utility of autofluorescence as an adjunct to routine cytology as it is simple, rapid, and cost-effective screening technique especially in developing countries where tuberculosis is widely prevalent and shows continued presence of infection in the community. However, limited and cautious use of AF is necessary because of its increased false-positivity rate and subjective-biased errors. AF should be used in addition to other ancillary techniques.

## Figures and Tables

**Table 1:** Comparative chart of the results of detection of *Mycobacteria* by Ziehl-Neelsen, auramine-rhodamine stain, and autofluorescence in various types of needle aspirates and cytomorphological patterns in the present study.

Appearance of aspirate	ZN	AR	AF
Blood mixed material	08	15	20
Pus-like material	06	07	09
Cheesy white material	10	08	08
Clear fluid	00	01	01
Cytomorphological patterns			
Granulomatous	10	16	26
Caseating necrotising	12	11	08
Acute inflammation with granuloma	02	04	04

**Table 2:** Comparative chart of results of the detection of *Mycobacteria* by Ziehl-Neelsen, auramine-rhodamine stain, and autofluorescence.

	ZN	AR	AF
TP (true positive)	20	22	24
FN (false negative)	05	03	01
FP (false positive)	04	09	14
TN (true negative)	61	56	51

**Table 3:** Taking culture as a reference method, analysis of statistical values of Ziehl-Neelsen, auramine-rhodamine stain, and autofluorescence.

	ZN	AR	AF
Sensitivity	80.0%	88.0%	96.0%
Specificity	93.85%	86.15%	78.46%
Positive predictive value	83.33%	70.97%	63.16%
Negative predictive value	92.42%	94.42%	98.08%

## References

[1] Dandapat MC, Mishra BM, Dash SP, Kar PK (1990). Peripheral lymph node tuberculosis: a review of 80 cases. *British Journal of Surgery*.

[2] Lau SK, Kwan S, Lee J, Wei WI (1991). Source of tubercle bacilli in cervical lymph nodes: a prospective study. *Journal of Laryngology and Otology*.

[3] Pahwa R, Hedau S, Jain S (2005). Assessment of possible tuberculous lymphadenopathy by PCR compared to non-molecular methods. *Journal of Medical Microbiology*.

[4] Singh KK, Muralidhar M, Kumar A (2000). Comparison of in house polymerase chain reaction with conventional techniques for the detection of Mycobacterium tuberculosis DNA in granulomatous lymphadenopathy. *Journal of Clinical Pathology*.

[5] Rajan VS, Goh YS (1972). Intermittent chemotherapy in the treatment of tuberculosis cutis. A preliminary report. *British Journal of Dermatology*.

[6] Daniel TM (1990). The rapid diagnosis of tuberculosis: a selective review. *Journal of Laboratory and Clinical Medicine*.

[7] Annam V, Kulkarni MH, Puranik RB (2009). Comparison of the modified fluorescent method and conventional Ziehl-Neelsen method in the detection of acidfast bacilli in lymphnode aspirates. *CytoJournal*.

[8] Jain A, Bhargava A, Agarwal SK (2002). A comparative study of two commonly used staining techniques for acid fast bacilli in clinical specimens. *Indian Journal of Tuberculosis*.

[9] Wright CA, van Zyl Y, Burgess SM, Blumberg L, Leiman G (2004). Mycobacterial autofluorescence in papanicolaou-stained lymph node aspirates: a glimmer in the dark?. *Diagnostic Cytopathology*.

[10] Wright CA, Hesseling AC, Bamford C, Burgess SM, Warren R, Marais BJ (2009). Fine-needle aspiration biopsy: a fi rst-line diagnostic procedure in paediatric tuberculosis suspects with peripheral lymphadenopathy?. *International Journal of Tuberculosis and Lung Disease*.

[11] Wright CA, van der Burg M, Geiger D, Noordzij JG, Burgess SM, Marais BJ (2008). Diagnosing mycobacterial lymphadenitis in children using fine needle aspiration biopsy: cytomorphology, ZN staining and autofluorescence-making more of less. *Diagnostic Cytopathology*.

[12] Joshi P, Singh M, Bhargava A, Singh M, Mehrotra R (2012). Autofluorescence—an important ancillary technique for the detection of Mycobacterium tuberculosis: revisited. *Diagnostic Cytopathology*.

[13] Patiño S, Alamo L, Cimino M (2008). Autofluorescence of mycobacteria as a tool for detection of Mycobacterium tuberculosis. *Journal of Clinical Microbiology*.

